# PTC725, an NS4B-Targeting Compound, Inhibits a Hepatitis C Virus Genotype 3 Replicon, as Predicted by Genome Sequence Analysis and Determined Experimentally

**DOI:** 10.1128/AAC.01272-16

**Published:** 2016-11-21

**Authors:** Jason D. Graci, Stephen P. Jung, John Pichardo, Frederick Lahser, Xiao Tong, Zhengxian Gu, Joseph M. Colacino

**Affiliations:** a PTC Therapeutics, Inc., South Plainfield, New Jersey, USA; b Department of Infectious Diseases, Merck Research Laboratories, Kenilworth, New Jersey, USA; c TREK Therapeutics, Cambridge, Massachusetts, USA

## Abstract

PTC725 is a small molecule NS4B-targeting inhibitor of hepatitis C virus (HCV) genotype (gt) 1 RNA replication that lacks activity against HCV gt2. We analyzed the Los Alamos HCV sequence database to predict susceptible/resistant HCV gt's according to the prevalence of known resistance-conferring amino acids in the NS4B protein. Our analysis predicted that HCV gt3 would be highly susceptible to the activity of PTC725. Indeed, PTC725 was shown to be active against a gt3 subgenomic replicon with a 50% effective concentration of ∼5 nM. *De novo* resistance selection identified mutations encoding amino acid substitutions mapping to the first predicted transmembrane region of NS4B, a finding consistent with results for PTC725 and other NS4B-targeting compounds against HCV gt1. This is the first report of the activity of an NS4B targeting compound against HCV gt3. In addition, we have identified previously unreported amino acid substitutions selected by PTC725 treatment which further demonstrate that these compounds target the NS4B first transmembrane region.

## INTRODUCTION

Chronic hepatitis C virus (HCV) liver infection is a significant cause of morbidity and mortality worldwide, with an estimated 185 million infected individuals (1). Despite recent advances in the development of effective therapeutics to treat HCV infection (2), current treatment regimens are suboptimal for some patients, including treatment-experienced patients and those infected with difficult-to-treat HCV genotypes (gt's), for example, gt3 infection (3, 4).

Genotype 3 is the second most commonly reported HCV genotype after gt1, with an estimated 54 million people infected worldwide (1) and is associated with accelerated progression of liver fibrosis, a higher incidence and severity of steatosis and hepatocellular carcinoma, and increased mortality compared to that caused by other HCV genotypes (5–8). While responsive to pegylated interferon alfa combined with ribavirin, the sustained virologic response (SVR) rates for gt3 (68%) are lower than those for gt2 (74%) (9). HCV gt3 has also proven to be more difficult to treat with direct-acting antivirals (DAAs), including NS3/4A protease inhibitors, which have limited activity against this gt (10, 11). While treatment regimens with newer DAAs targeting the viral NS5A and NS5B proteins (often in combination with ribavirin) have achieved higher SVR rates in gt3 patients, these DAAs remain suboptimal, particularly in treatment-experienced patients and patients with liver cirrhosis (12–17).

**FIG 1:**
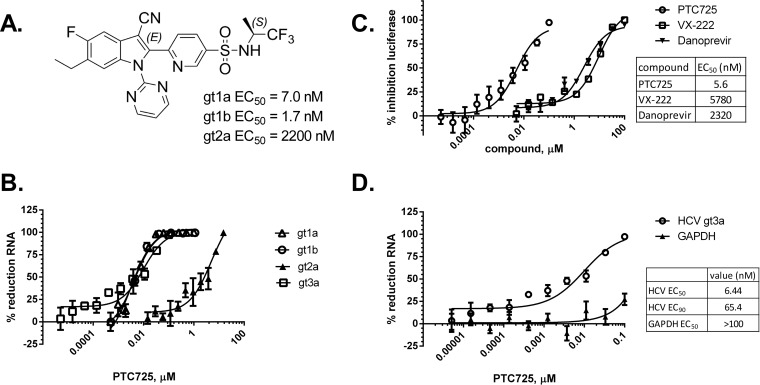
PTC725 is a potent and selective inhibitor of an HCV genotype 3a replicon. (A) Structure of PTC725 [(*S*)-6-(3-cyano-6-ethyl-5-fluoro-1-(pyrimidin-2-yl)-1*H*-indol-2-yl)-*N*-(1,1,1-trifluoropropan-2-yl)pyridine-3-sulfonamide)] and activity against the indicated HCV genotypes. (B) PTC725 inhibits HCV gt1a, gt1b, and gt3a but has substantially reduced activity against gt2a. qRT-PCR was used to measure HCV RNA 72 h after compound treatment. (C) Luciferase activity in S52/SG-Feo(SH) after 72 h treatment with PTC725 or comparator inhibitors. (D) qRT-PCR of S52/SG-Feo(SH) lysates after 72 h of treatment with PTC725, using probe/primer sets specific for HCV gt3 or human GAPDH. For panels B, C, and D, the results of one representative experiment are plotted as means ± the SEM (*n* = 2). Summary data are provided in Table 3.

PTC725 (Fig. 1A) was identified as a potent small molecule inhibitor of HCV gts 1a and 1b replicon RNA replication (50% effective concentration [EC_50_] = 1 to 7 nM) but not of infectious gt2 virus (EC_50_ = 2,200 nM) (18). *De novo* selection of resistance in a gt1 replicon revealed amino acid substitutions in the NS4B protein, particularly H94R, F98C/L, and V105M, that conferred 16- to 300-fold resistance to PTC725. The lack of activity against gt2 virus was consistent with the high frequency of L98 in reported gt2 sequences, including the JFH-1 virus used in many *in vitro* studies. Notably, other reported NS4B-targeting compounds have shown similar resistance and activity profiles (19–23).

HCV NS4B is a 27-kDa integral transmembrane protein produced from cleavage of the HCV genome-encoded polyprotein precursor (reviewed in references 24 and 25). Although the precise function(s) of NS4B in HCV replication is still under investigation, the protein appears to direct the formation of viral replication complexes from the host endoplasmic reticulum membrane and the recruitment of other HCV nonstructural proteins (26–30). Furthermore, NS4B has been reported to have NTPase and adenylate kinase activities and to bind HCV RNA (31, 32). Modeling and experimental results suggest that NS4B has four transmembrane domains (TM1 to TM4), with a potential fifth membrane-spanning segment (TMX) (33, 34). Amino acid substitutions conferring resistance to PTC725 and other reported NS4B targeting compounds are localized to the first predicted transmembrane domain, TM1.

Due to the marked difference in the activity of PTC725 against HCV gt1 compared to that against gt2, we sought to understand further the genotype-specific response to this compound. Here, we compare known PTC725 resistance-conferring mutations to an HCV sequence database to predict the activity of PTC725 against HCV gt3, and we confirm the activity by testing against an HCV gt3 subgenomic replicon. Consistent with the central role of the NS4B TM1 region in the activity of these compounds, sequencing of gt3 replicons resistant to PTC725 identified mutations encoding amino acid changes in NS4B similar but not identical to those observed in gt1 replicons selected for resistance to PTC725.

## MATERIALS AND METHODS

### Compounds.

PTC725 [(*S*)-6-(3-cyano-6-ethyl-5-fluoro-1-(pyrimidin-2-yl)-1*H*-indol-2-yl)-*N*-(1,1,1-trifluoropropan-2-yl)pyridine-3-sulfonamide)] (18) was synthesized at PTC Therapeutics, Inc. (South Plainfield, NJ). Danoprevir and VX-222 (lomibuvir) were purchased from Selleck Chemicals (Houston, TX).

### HCV replicon assay.

Inhibition of HCV gt1a, gt1b, and gt2a RNA replication by PTC725 was evaluated by quantitative reverse transcription-PCR (qRT-PCR) as previously described (18, 35). The HCV gt3a replicon S52/SG-Feo(SH) (36) was licensed from Apath, LLC (New York, NY). This replicon is derived from a full-length HCV gt3 cDNA in which amino acids 20 to 1032 were replaced by a chimeric gene encoding firefly luciferase fused in frame with neomycin phosphotransferase, followed by the encephalomyocarditis virus internal ribosome entry site (IRES), which directs translation of the downstream NS3-NS5B region. Huh7.5 cells bearing the S52/SG-Feo(SH) replicon were maintained in Dulbecco modified Eagle medium containing 10% fetal bovine serum, 0.01 mM nonessential amino acids, and 0.5 mg/ml G418 (all reagents from Invitrogen, Carlsbad, CA). For compound activity assays, replicon-bearing cells were seeded at 5,000 cells/well in 96-well plates in growth medium without G418 and treated with various concentrations of PTC725 in dimethyl sulfoxide (DMSO; final concentration of DMSO, 0.5%). The firefly luciferase activity was quantified 3 days after dosing using steadylite plus (Perkin-Elmer, Waltham, MA), and the percent inhibition was determined by comparison to vehicle-treated cells. Alternatively, cells were harvested 3 days after dosing, and HCV RNA was quantified by real-time qRT-PCR using primers and a probe specific to the gt3 IRES region in the 5′ untranslated region (forward, 5′-ACGCGGAAAGCGCCTAGCCAT-3′; reverse, 5′-TACTCACCGGTTCCGCAGA-3′; probe, 5′-6FAM-TCCTGGAGGCTGCACGACACTCGT-TAMRA-3′ [Applied Biosystems, Foster City, CA]). Human GAPDH (glyceraldehyde-3-phosphate dehydrogenase) RNA was monitored as an endogenous control for selectivity using a predeveloped TaqMan assay (Applied Biosystems). RNA was quantified on an Applied Biosystems 7900HT. PCR replicates were used for each biological sample. The percent inhibition of HCV and GAPDH RNA production was determined by comparison to vehicle-treated cells. The EC_50_s were determined by nonlinear regression using GraphPad Prism (La Jolla, CA).

### Frequency of selected resistant colonies.

Huh7.5 cells bearing the S52/SG-Feo(SH) replicon were plated at 10^6^ cells per 100-mm culture dish under G418 selection (0.5 mg/ml) and treated with PTC725 or danoprevir at the indicated concentrations. As a control for compound toxicity, Huh7 cells were treated with compounds in the absence of G418. Plates were incubated for 3 weeks with medium changed twice weekly. After 3 weeks, the plates were washed with phosphate-buffered saline and stained with 2% Gentian violet aqueous solution (Ricca Chemical Company, Arlington, TX). Cell colonies were counted on each plate, and the frequency of resistance was determined as the number of observed colonies/the number of cells plated.

### *De novo* selection of resistance.

Replicon-bearing cells were cultured at subconfluence with fixed concentrations of PTC725 in the presence of G418 at 0.5 mg/ml. In parallel, replicon-bearing cells cultured in the absence of PTC725 were used as a mock selection control for the appearance of nonspecific mutations. Cell culture medium was replenished with fresh medium containing the appropriate concentration of PTC725 every 3 to 4 days until visible colonies formed, at which time the cells were harvested by trypsinization and seeded into T-75 flasks. The cell growth rate was monitored as an indicator of replicon resistance, and the susceptibility of the selected replicons to PTC725 was evaluated by quantification of the firefly luciferase activity as described above. RNA was isolated from resistant cells using Ambion PureLink RNA minikit (Thermo Fisher Scientific, Waltham, MA), and cDNA was generated using a SuperScript III kit (Invitrogen) using random hexamers as primers. A 1.6-kb DNA containing the NS4B coding region was generated from the cDNA using Platinum *Taq* DNA polymerase (Invitrogen) and the primers 5′-CGG TTG TTC TCT GTG AGT GC-3′ and 5′-CCG GTG GTG TAC TCA TTG-3′ and purified by using an Invitrogen PureLink Quick PCR purification kit. Fragments were cloned into the pCR2.1-TOPO vector using TOPO TA cloning kit and cloned into TOP10 chemically competent Escherichia coli (Invitrogen). Colonies were selected on Luria-Bertani plates with ampicillin at 0.1 mg/ml. Individual clones were picked and then sequenced by Genewiz, Inc. (South Plainfield, NJ). Sequences were analyzed using Vector NTI (Invitrogen).

### Data analysis.

Data were plotted with GraphPad Prism (La Jolla, CA) and are presented as means ± the standard errors of the mean (SEM). A sigmoidal dose-response curve with a variable-slope regression curve was generated to determine the EC_50_s.

## RESULTS

### Analysis of NS4B variants by HCV genotype.

Previous *de novo* selection of replicons resistant to PTC725 (Fig. 1A) identified four resistance-associated variants (RAVs) at three positions in the TM1 region of the HCV NS4B protein (H94R, F98C/L, and V105M) (18). Using a typical model of 3.5 amino acids/turn, these amino acid substitutions occur on the same face of TM1. During lead optimization of this compound series, further resistance selection using structural analogs of PTC725 (see Fig. S1 in the supplemental material) identified a number of additional RAVs (Table 1) that confer resistance to PTC725 in the gt1b (Con1) replicon. We identified substitutions at S59C/G and L109I/R, as well as an additional substitution at position 105 (V105L). Of note, L109 is predicted to occur on the same face of the TM1 helix as H94, F98, and V105. S59 is located N-terminal to TM1 in an α-helical segment that has been reported to associate and possibly traverse cell membranes (37). Each of the nine identified RAVs were singly engineered into gt1b replicons as previously described (18) and shown to confer 6.7- to 130-fold resistance to PTC725 (Table 1), with similar resistance profiles observed for structurally related analogs of PTC725.

**TABLE 1:** Amino acid changes conferring resistance to PTC725

Amino acid	Con1 gt1b replicon	PTC725 resistance-conferring residue(s)	Fold resistance to PTC725
59	S	C/G	160/80
94	H	R	16
98	F	C/L	300/57
105	V	L/M	6.7/100
109	L	I/R	10/130

We hypothesized that the poor activity of PTC725 against gt2 is due to the high frequency of L98 found in the NS4B of gt2 isolates, including in the JFH-1 virus (18). To understand further the expected susceptibility of various gt's to PTC725, we analyzed all available NS4B sequences in the Los Alamos hepatitis C sequence database (38) to determine the amino acid distribution at five known resistance loci residues for each gt (Table 2). A number of RAVs were identified in gt1 populations, but only at a low frequency (≤2.1% of reported sequences), an observation consistent with the potent inhibition of gt1 replicons by PTC725. The amino acid variants L98, L105, and I109 were highly prevalent (>98%) in HCV gt2, providing an explanation for the lack of activity of PTC725 against the JFH-1 virus (18). The amino acid L98 is also highly prevalent in HCV gt6, predicting that PTC725 will have poor activity against this gt.

**TABLE 2:** NS4B amino acid frequency at resistance loci by HCV genotype

gt	Distribution (%) of PTC725 resistance-related mutations^*a*^					No. of sequences
	aa 59	aa 94	aa 98	aa 105	aa 109	
gt1	0	0	L (2)	L (2.1), M (0.1)	I (0.2)	1,013
gt2	0	0	L (100)	L (98)	I (98)	61
gt3	0	0	0	0	0	118
gt4	0	0	0	0	I (100)	44
gt5	0	0	0	0	I (100)	3
gt6	0	0	L (100)	L (6)	I (1)	62

a aa, amino acid.

Although only a limited number of sequences were available, gt4 and gt5 did not appear to have a significant frequency of known resistance substitutions at amino acid positions 59, 94, 98, or 105. However, the available sequences invariably encoded I109, a resistance-conferring substitution also found at high prevalence in gt2. Notably, no known RAVs were identified in 118 reported gt3 sequences. Therefore, we hypothesized that PTC725 would be highly active against HCV gt3.

### PTC725 inhibition of an HCV gt3 subgenomic replicon.

PTC725 was tested for activity in Huh7.5 cells bearing the S52/SG-Feo(SH) replicon that encodes a chimeric firefly luciferase reporter gene in place of the HCV structural proteins (36). The inhibitory activity of PTC725 was determined 3 days after dosing by quantifying the firefly luciferase activity, which is proportional to the HCV RNA level (Fig. 1C), or replicon RNA levels via qRT-PCR using primers and probe specific to the HCV gt3 genomic RNA and human GAPDH RNA as an endogenous control for selectivity (Fig. 1B and D). As predicted, PTC725 was highly active against the gt3 replicon with an EC_50_ of ∼5 nM using either assay endpoint (Table 3), similar to the potency observed against the HCV gt1 replicon. Consistent with previous data showing high selectivity with respect to cytotoxicity, PTC725 treatment resulted in little or no inhibition of cellular GAPDH RNA production at the concentrations tested (Fig. 1D, Table 3). Danoprevir, an HCV NS3/4A protease inhibitor, had EC_50_s of 3.6 and 2.6 μM, as determined by luciferase assay and qRT-PCR, respectively, an observation consistent with prior reports indicating low susceptibility of HCV gt3 replicons to this compound (36). VX-222 (lomibuvir), a nonnucleoside HCV NS5B polymerase inhibitor, was similarly a poor inhibitor of gt3, as has been previously demonstrated for polymerase thumb site II inhibitors as a class (39), with EC_50_s of 5.8 and 4.8 μM, as determined by luciferase assay and qRT-PCR, respectively. Therefore, a lack of preexisting resistance mutations in the HCV gt3 NS4B coding sequence predicted the susceptibility of this genotype to inhibition by PTC725.

**TABLE 3:** PTC725 activity and selectivity in gt3 replicon cells

Inhibition of:	Assay	EC_50_ (nM) ± SEM^*a*^
Genotype 3a replicon	Luciferase	5.4 ± 2.4
Genotype 3a replicon RNA	qRT-PCR	5.3 ± 2.1
GAPDH RNA	qRT-PCR	>100

a Data represent the means of at least three independent experiments.

### Frequency of selected resistant colonies.

**FIG 2:**
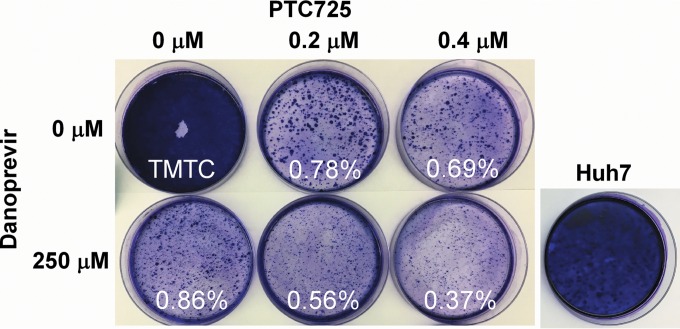
PTC725 and danoprevir in combination suppress the emergence of HCV gt3 replicon resistance. HCV gt3 replicon-bearing cells were treated with PTC725 or danoprevir alone or in combination under G418 selection. The frequency (%) of drug-resistant replicons was calculated as the number of observed colonies/the number of cells plated. The rightmost panel shows Huh7 cells exposed to 250 μM danoprevir and 0.4 μM PTC725 for an equivalent length of time as a control for compound toxicity. TMTC, too many to count.

The frequency of colonies selected for resistance to PTC725 was assessed in S52/SG-Feo(SH) replicon-bearing cells. Cells under G418 selection were cultured in the presence of PTC725 alone, danoprevir alone, or both of these agents combined. PTC725 was used at approximately 40× and 80× its EC_50_, while danoprevir was used at 100× its EC_50_. The frequency of drug-resistant colonies of replicon-bearing cells was 0.69% when exposed to 80× EC_50_ PTC725 and 0.86% with a 100× EC_50_ of danoprevir (Fig. 2). The combination of PTC725 (80× EC_50_) and danoprevir (100× EC_50_), however, reduced the frequency of replicon-resistant colonies to 0.37%. These data indicate that PTC725 in combination with an NS3/4A protease inhibitor suppresses the selection of HCV replicon resistance.

### *De novo* selection of resistance.

**FIG 3:**
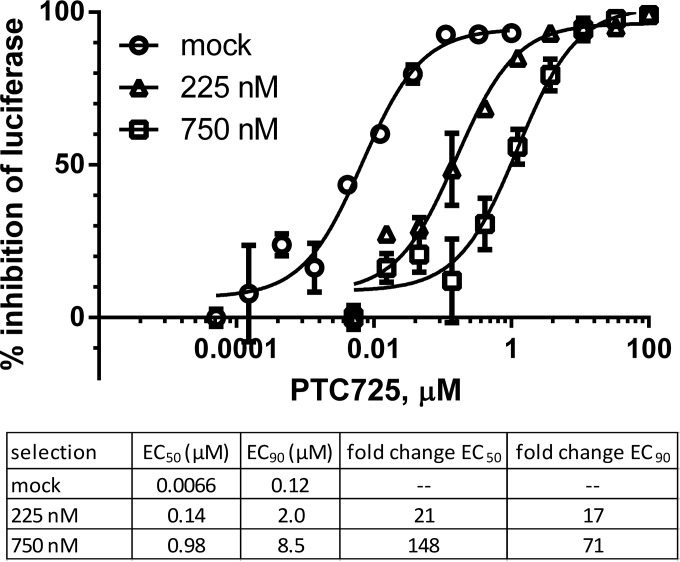
HCV gt3 replicon populations selected with PTC725 exhibit reduced susceptibility to PTC725. HCV gt3 replicon cell populations selected with various concentrations of PTC725 were treated with PTC725 in dose response in the absence of G418. After 72 h, the luciferase activity was quantified and compared to that in the vehicle-treated control. Data are plotted as means ± the SEM (*n* = 2).

*De novo* selection of resistance was undertaken by passaging S52/SG-Feo(SH) replicon-bearing cells in the presence of various concentrations of PTC725 (Table 4). Cells were selected with 0.5 mg/ml G418, with replenishment of medium and compound twice per week and maintenance at <70% confluence, until growth rate and luciferase expression normalized to that of mock-treated cells (see Fig. S2 in the supplemental material). Selection at the highest concentration of PTC725 (2,250 nM) yielded no recoverable cells. Recovered cell populations were tested for replicon susceptibility to PTC725 by quantifying the luciferase activity (Fig. 3) and found to have acquired 20- and 150-fold resistances to PTC725 when selected with 225 and 750 nM PTC725, respectively. Total RNA was isolated from selected cells and used to prepare cDNA, which was then cloned and transformed into E. coli as previously described (18).

**TABLE 4:** Identified HCV genomic mutations and amino acid substitutions after *de novo* selection for PTC725 resistance

PTC725 treatment (no. of clones)^*a*^	PTC725 EC_50_ (nM)^*b*^	Frequency of nucleotide mutation (amino acid substitution)^*c*^							
		C190A (L64I)	T253G (S85A)	A262T (S88C)	T290A (F97Y)	TT289CA (F97H)	G313A (V105I)	G327T (L109F)	T326G (L109W)
Mock (19)	6.6	0 (0)	0 (0)	0 (0)	0 (0)	0 (0)	0 (0)	0 (0)	0 (0)
225 nM (17)	140	2 (12)	1 (6)	0 (0)	0 (0)	0 (0)	3 (18)	**11 (65)**	0 (0)
750 nM (28)	980	0 (0)	0 (0)	1 (4)	**16 (57)**	1 (4)	0 (0)	7 (25)	3 (11)
2,250 nM^*d*^	NA	NA	NA	NA	NA	NA	NA	NA	NA

a The number of clones for which NS4B was sequenced is indicated in parentheses.

b The EC_50_ values were determined for the pooled selected replicon population for each condition using the luciferase activity as an endpoint after 72 h of treatment (*n* = 2).

c The total number of colonies for each variant selected is reported; the frequency (%) in indicated in parentheses. The most frequently observed variant under each condition is indicated in boldface.

d NA, not applicable (no viable replicon cells were recovered under this selection condition).

Sequencing of the HCV NS4B-coding sequence revealed mutations encoding substitutions in the NS4B protein (Table 4; see Fig. S3 in the supplemental material). At selection with 225 nM PTC725, the predominant amino acid alteration identified was L109F in 11 of 17 clones sequenced. This result was not surprising considering L109I/R substitutions were identified as resistance-conferring alterations in gt1, resulting in 10- to 130-fold resistance to PTC725 (Table 1). Three of seventeen clones contained V105I, whereas alternate substitutions at this position (V105L/M) were identified in gt1. The remaining clones contained amino acid alterations N-terminal to TM1, at L64I (2 of 17 clones) and S85A (1 of 17 clones).

In contrast, gt3 replicons selected with 750 nM PTC725 predominantly displayed a substitution at amino acid F97 (Y/H) in 17 of 28 sequenced clones. In the gt1b Con1 replicon, position 97 is L rather than F; however, the predominant substitutions conferring resistance to PTC725 in gt1 were found at the adjacent residue F98 (see Fig. S3 in the supplemental material). In 10 clones, substitutions of L109 were identified (F/W), similarly to those observed with selection at 225 nM PTC725. An additional substitution was observed at S88C, located slightly N-terminal to TM1.

## DISCUSSION

HCV, like many RNA viruses, exhibits considerable genetic diversity and is presently classified into 7 genotypes comprising 67 confirmed subtypes (40). Clinical experience demonstrates that different genotypes can have differential responses to therapeutic intervention. In some cases, as with some narrow-spectrum HCV NS3/4A protease inhibitors, drugs may be active against one genotype but inactive against others due to critical sequence divergence in the drug binding site (39). PTC725 was previously shown to be an effective inhibitor of HCV gt1 replicon replication but with greatly decreased potency against HCV gt2 (18). Here, we demonstrate that PTC725 can be targeted to specific HCV genotypes based on genomic sequencing data and that it can act in concert with a protease inhibitor to restrict the selection of resistance.

In recent years, a number of small molecules targeting HCV NS4B have been described. Many of these have selected for resistance mutations in the coding region of NS4B residues 94 to 109 in gt1 HCV, a region predicted as the first of four transmembrane α-helices (19–23). Furthermore, the most prominent substitutions (at residues 94, 98, 105, and 109) lie on the same face of the α-helix according to the standard model based on 3.5 amino acids per turn. We found that similar substitutions occur in PTC725-resistant gt3 replicons (see Fig. S3 in the supplemental material), most prominently at L109, but also at V105. Interestingly, whereas substitutions at F98 are a major resistance determinant in HCV gt1, PTC725-resistant HCV gt3 replicons exhibited substitutions predominantly in F97, including F97Y and F97H.

We also report mutations encoding amino acid substitutions N-terminal to the first transmembrane helix (TM1), specifically S59C/G in the HCV gt1b replicon, as conferring resistance to PTC725. In HCV gt3 replicons, minor substitutions were also identified N terminal to TM1 after *de novo* selection, including L64I, S85A, and S88C. The magnitude of resistance to PTC725 and the effect on HCV fitness are yet to be determined for these substitutions; however, an HCV NS4B-targeting compound discovered through encoded library technology was found to exhibit 3-fold-less potency against replicons containing S85A (23). Notably, S85 and S88 are located in the region postulated to be a fifth membrane-spanning domain (TMX), suggesting that NS4B-targeting compounds such as PTC725 may affect the interaction of these two transmembrane regions (33, 34).

To date, the structure of NS4B remains largely unknown and the precise functions of NS4B in the HCV life cycle remain poorly defined. However, computational modeling and experimental evidence suggest that PTC725 targets the membrane-spanning region of NS4B known as TM1. A wide range of mutations conferring resistance to the activity of NS4B-targeting compounds has been described by us and others, with the most significant mutations encoding amino acid substitutions spanning the length of the proposed TM1 helix and others located upstream in the putative TMX region. These data may suggest a critical interaction between TM1 and TMX that is disrupted by NS4B-targeting compounds but can be restored by a variety of amino acid substitutions stabilizing the interaction or disrupting the small molecule binding surface(s). However, a physical interaction between TM1 and TMX has yet to be demonstrated, and the membrane-spanning properties of TMX remain unclear. Others have hypothesized that an interaction between TM1 and TM2 is essential for viral replication based on defective replication of JFH-1 gt2a virus containing gt1b NS4B TM1 or TM2 regions (41). Further understanding of NS4B structure and its architecture will illuminate the role of this protein in viral replication.

Recently, an NS4B-targeting compound (GSK8853) structurally unrelated to PTC725 was reported to inhibit HCV gt1a replication in a humanized mouse model and elicited resistance mutations *in vivo* which were distinct from those identified in resistant replicons *in vitro*, i.e., those encoding substitutions at positions 94, 98, and 105 (42). RAVs were identified *in vivo* at N56I and N99H and were confirmed to confer resistance in genetically engineered replicons. Interestingly, N99H conferred a 4,600-fold shift in potency against GSK8853 and is adjacent to the resistance substitutions found at F98 for this molecule and other NS4B-targeting compounds in cell culture. Our finding that amino acid substitutions throughout the TM1 region of NS4B occur during selection of gt3 replicons with PTC725 implicates this region in the activity of PTC725 and other small molecules that have been reported to target NS4B.

We demonstrate that PTC725 is a highly potent inhibitor of HCV gt1 and gt3 replicons. Based on HCV sequence analysis, we predict that this compound would have limited activity against HCV gt's 2, 4, 5, and 6, although the lack of activity has only been shown experimentally for gt2 (18). In the present study, we utilized a sequence database of HCV genotypes to predict susceptibility to an antiviral compound with a known resistance profile. Further understanding of the genetic composition of various HCV genotypes and subtypes may be useful in predicting the optimal drug therapies to treat infections caused by each. Given the less-than-optimal efficacy of current treatments for HCV gt3 infection, PTC725 may represent a potential addition to combination therapy “cocktails” for the treatment of HCV in patients who respond poorly to current therapies.

## Supplementary Material

Supplemental material
