# Supplementary material for: PTC725, an NS4B-Targeting Compound, Inhibits a Hepatitis C Virus Genotype 3 Replicon, as Predicted by Genome Sequence Analysis and Determined Experimentally
Source: Antimicrob Agents Chemother. 2016 Nov 21;60(12):7060–6. doi: 10.1128/AAC.01272-16 (PMC5118984; doi:10.1128/AAC.01272-16)
Supplement: Supplemental material [file supp_60_12_7060__index.html]

Supplemental material 

# PTC725, an NS4B-Targeting Compound, Inhibits a Hepatitis C Virus Genotype 3 Replicon, as Predicted by Genome Sequence Analysis and Determined Experimentally

## Supplemental material

- Supplemental file 1 -

  Fig. S1-S3

  PDF, 276K
