# Supplementary material for: PTC725, an NS4B-Targeting Compound, Inhibits a Hepatitis C Virus Genotype 3 Replicon, as Predicted by Genome Sequence Analysis and Determined Experimentally
Source: Antimicrob Agents Chemother. 2016 Nov 21;60(12):7060–6. doi: 10.1128/AAC.01272-16 (PMC5118984; doi:10.1128/AAC.01272-16)
Supplement: Supplemental material [file AAC.01272-16_zac012165712so1.pdf]

PTC725, an NS4B-targeting compound, inhibits an HCV genotype 3 replicon, as predicted by genome sequence analysis and determined experimentally

Jason D. Graci, Stephen P. Jung, John Pichardo, Frederick Lahser, Xiao Tong, Zhengxian Gu, and Joseph M. Colacino

Supplemental Information

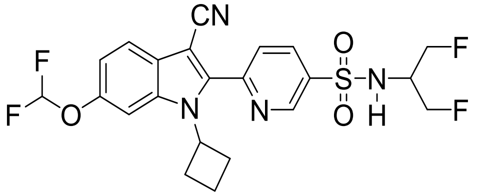

S59G/C, V105L

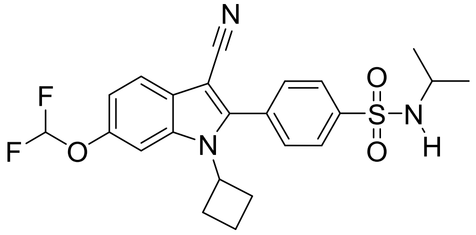

L109I/R

Figure S1. Earlier lead compounds revealed additional PTC725 resistance-associated variants (RAVs) after de novo selection. The first compound has been previously reported as compound 2 in (1). Compounds were synthesized at PTC Therapeutics, Inc.

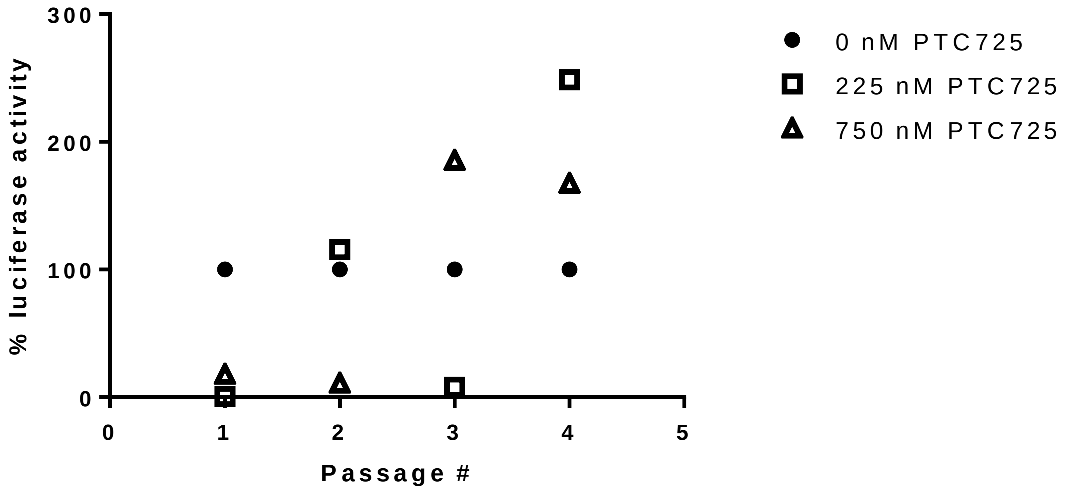

Figure S2. Relative fitness of PTC725-selected replicon cells. Colonies of PTC725-selected HCV gt3 replicon cells were harvested, pooled, and serially passaged. A luciferase assay was used to quantify replicon replication. Data are plotted as percent luciferase activity compared to mock treated control. By 4 passages post-harvest, cells regained full HCV replication capacity as compared to mock-treated cells.

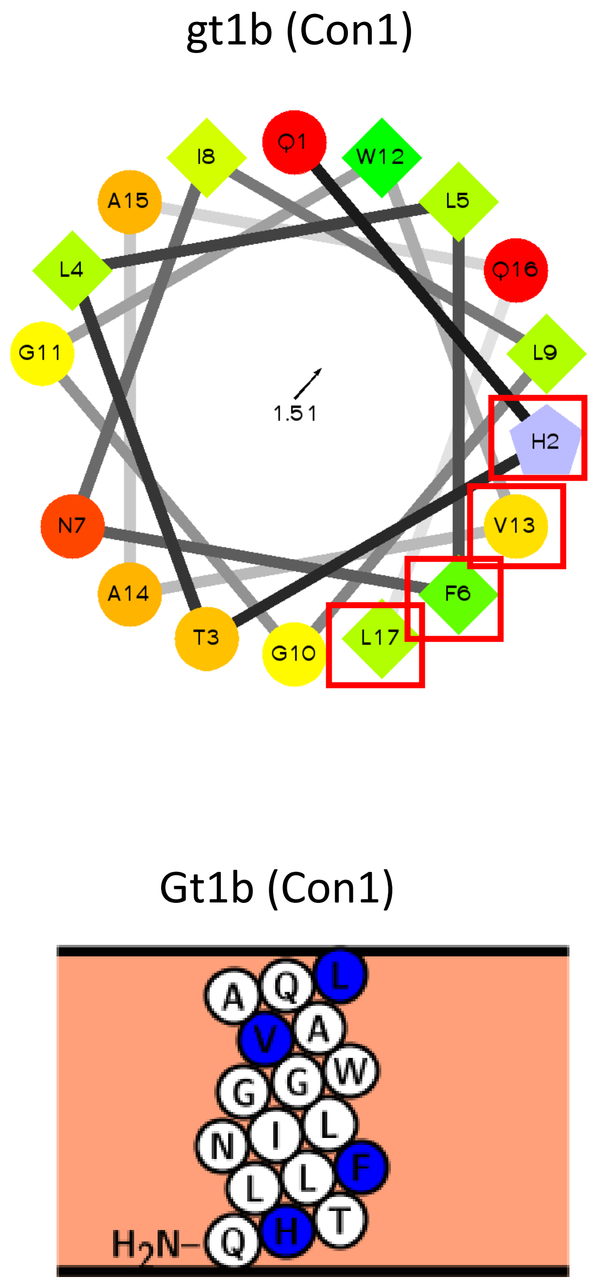

Figure S3. Predicted topology of RAVs found in NS4B TM1. (A) Helical wheel diagrams were generated using EMBOSS pepwheel hosted at http://www.tcdb.org/progs/?tool=pepwheel. The boundaries of the transmembrane helix are based on prior work (2), where TM1 spans NS4B residues 93-109, notated here as residues 1-17. Known PTC725 RAVs for gt1b (left) and substitutions selected by PTC725 in gt3a (right) are indicated by red boxes. (B) Transmembrane diagram was created using Protter hosted at http://wlab.ethz.ch/protter/start/ (3). RAVs identified for gt1b and substitutions selected for in gt3a are highlighted in blue.

REFERENCES

1. **Zhang, N., X. Zhang, J. Zhu, A. Turpoff, G. Chen, C. Morrill, S. Huang, W. Lennox, R. Kakarla, R. Liu, C. Li, H. Ren, N. Almstead, S. Venkatraman, F. G. Njoroge, Z. Gu, V. Clausen, J. Graci, S. P. Jung, Y. Zheng, J. M. Colacino, F. Lahser, J. Sheedy, A. Mollin, M. Weetall, A. Nomeir, and G. M. Karp**. 2014. Structure-activity relationship (SAR) optimization of 6-(indol-2-yl)pyridine-3-sulfonamides: identification of potent, selective, and orally bioavailable small molecules targeting hepatitis C (HCV) NS4B. J.Med.Chem. **57**:2121-2135. doi:10.1021/jm401621g [doi].

2. **Boleti, H., D. Smirlis, G. Dalagiorgou, E. F. Meurs, S. Christoforidis, and P. Mavromara**. 2010. ER targeting and retention of the HCV NS4B protein relies on the concerted action of multiple structural features including its transmembrane domains. Mol.Membr.Biol. **27**:50-74.

3. **Omasits, U., C. H. Ahrens, S. Muller, and B. Wollscheid**. 2014. Protter: interactive protein feature visualization and integration with experimental proteomic data. Bioinformatics. **30**:884-886. doi:btt607 [pii];10.1093/bioinformatics/btt607 [doi].
